# Synchronous multiple primary cancers involving rectal cancer and pelvic classical hodgkin lymphoma: the first case report

**DOI:** 10.3389/fonc.2023.1295533

**Published:** 2023-11-24

**Authors:** Shiyue Liu, Hong Li, Youhong Dong, Dongdong Zhang

**Affiliations:** ^1^ Department of Oncology, Xiangyang No.1 People`s Hospital, Hubei Univeristy of Medicine, XiangYang, Hubei, China; ^2^ Department of Rehabilitation Medicine, Xiangyang No.1 People`s Hospital, Hubei Univeristy of Medicine, XiangYang, Hubei, China

**Keywords:** rectal cancer, Hodgkin lymphoma, multiple primary cancers, diagnostic evaluation, surgical treatment

## Abstract

Multiple primary cancers (MPC) are characterized by the presence of synchronous and metachronous occurrence of two or more distinct histological tumor types. In this study, an exceptional clinical case was presented, demonstrating the coexistence of rectal adenocarcinoma and pelvic classical Hodgkin lymphoma (cHL). A 65-year-old male patient with a 2-year history of persistent mucous bloody stools was admitted to our hospital. Colonoscopy and subsequent biopsy confirmed the diagnosis of rectal adenocarcinoma. The patient underwent laparoscopic abdominoperineal resection of the rectum and regional lymph node dissection. Postoperative histopathological analysis not only substantiated the presence of rectal adenocarcinoma, but also unexpectedly identified pelvic lymph nodes harboring the features of cHL.

## Introduction

The incidence of multiple primary cancers (MPC) has shown an upward trend in the general population (1), accounting for approximately 1-16% of all cancer cases (2, 3). The etiology of MPC was found to be multifactorial, involving factors, such as genetic predisposition, environmental exposures, and lifestyle choices. The presence of MPC complicates the diagnostic process, treatment plan formulation, and prognosis assessment, and no unified standard was established. Nonetheless, accurate diagnosis of each specific type and stage of MPC is crucial, and developing the most effective treatment plan for each specific type of tumor is vital for patients’ prognosis.

Rectal cancer is one of the most common and detrimental digestive system tumors (4). The incidence of synchronous double primary malignancies in rectal cancer patients is relatively low, and the reported cases of double primary malignancies in rectal cancer most commonly occurred concurrently with other digestive system tumors, such as esophageal cancer (5), gastric cancer (6), colon cancer (7), and intrahepatic cholangiocarcinoma (8). Lymph nodes are the most frequent site of colorectal cancer (CRC) metastasis, and they are identified in approximately 30% of rectal cancer patients at the time of diagnosis (9). However, the simultaneous occurrence of lymph node metastasis in rectal adenocarcinoma and lymphoma is exceedingly rare. In this report, an intriguing case was presented who was diagnosed with rectal adenocarcinoma and cHL simultaneously.

## Case presentation

**Figure 1:**
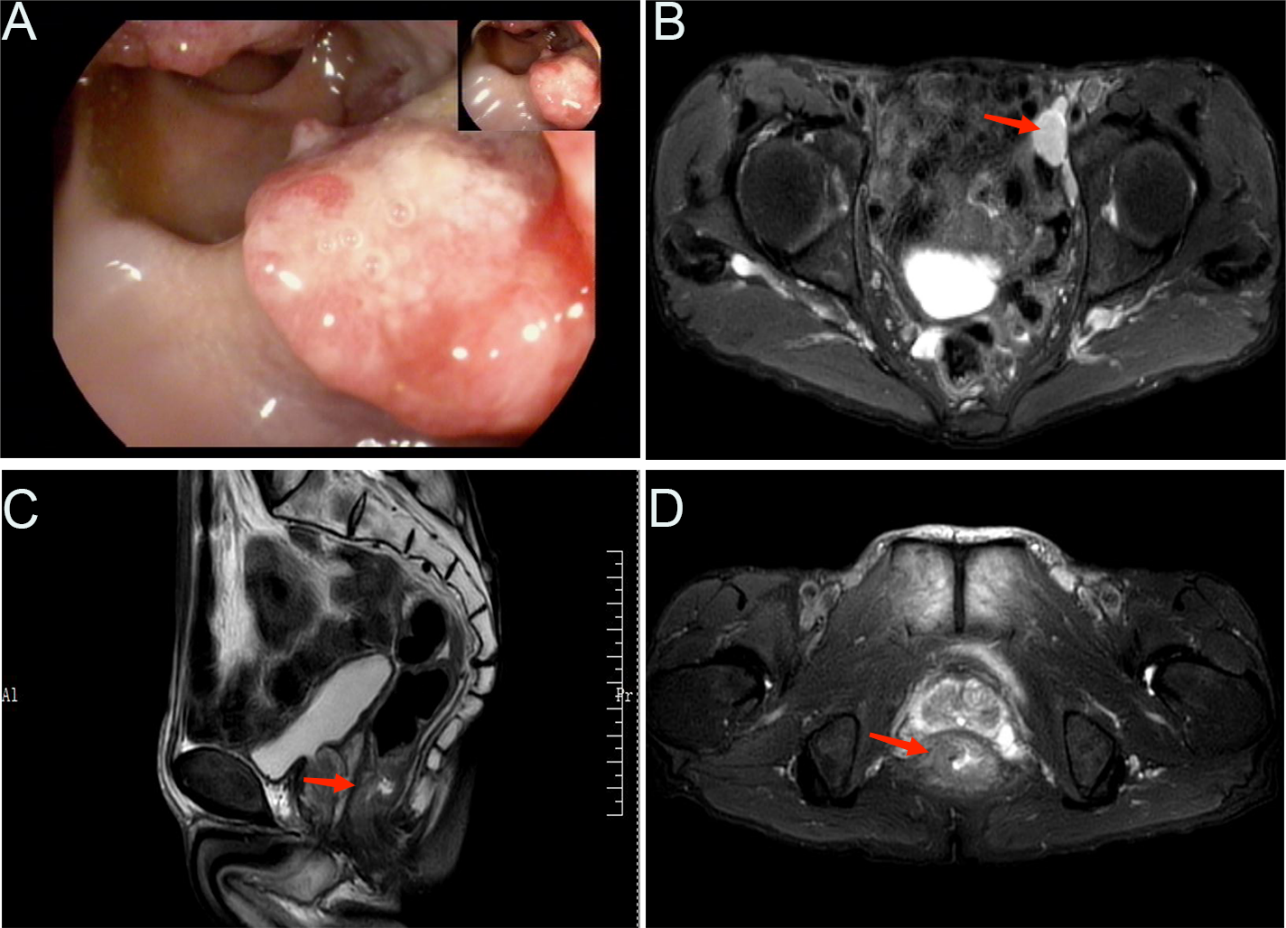
Radiological examination findings. **(A)** Representative image showing the colonoscopic view of the colon. **(B)** MRI findings, with an arrow indicating an enlarged lymph node adjacent to the left iliac artery. MRI findings of a rectal mass in both sagittal **(C)** and coronal **(D)** views, as indicated by the arrows.

A 65-year-old male patient was admitted to our hospital with a complaint of mucopurulent bloody stools persisting for 2 years and anal pain for 2 months. The patient reported that approximately 3-4 times per day, he experienced increased bowel movements accompanied by a sense of fullness and discomfort, without any apparent precipitating factors. Initially, he paid little attention to these symptoms and did not receive any specific treatment. However, the symptoms did not improve significantly over time, and the sense of fullness and discomfort gradually worsened. Over the past 2 months, the patient started experiencing intermittent nocturnal stabbing pain in the anal region. Digital rectal examination revealed a cauliflower-like mass in the K-C position, palpable on the anterior wall of the rectum, located approximately 2cm from the anal verge. The mass exhibited a hard texture, had infiltrated about half of the intestinal wall, showed limited mobility, and was associated with blood-stained gloves. Colonoscopy findings indicated a rectal mass, highlighting the necessity of biopsy for further evaluation (**Figure 1A**). Pathological analysis confirmed rectal adenocarcinoma.

Further imaging studies included abdominal and pelvic magnetic resonance imaging (MRI) (**Figures 1B–D**), which revealed the following results: 1) No significant abnormalities were observed on the plain scan of the upper abdomen; 2) Changes in the lower segment of the rectum were suggestive of a tumor, with a relatively irregular margin, and multiple enlarged lymph nodes adjacent to the left iliac vessels. Upon comprehensive assessment, the patient demonstrated clear indications for surgery without any contraindications. Intraoperatively, the tumor was observed to be located 2 cm from the anal verge. Accordingly, the patient underwent the Miles procedure, which involved laparoscopic-assisted abdominoperineal resection of the rectum, along with mesenteric lymph node dissection, left lateral lymph node dissection, sigmoid colostomy, and abdominal drainage.

Notably, postoperative pathological examination of the rectal mass indicated invasive ulcerative moderately differentiated adenocarcinoma, measuring 4.0 × 3.5 × 1.0 cm^3^, infiltrating from the muscularis propria to the subserosa layer of the bowel wall. No definite intravascular cancer emboli or neural invasion were observed. The resection margins of the rectum, anal skin, and mesentery were free of tumor involvement. Surrounding mucosal tissue showed chronic inflammation. Lymph node dissection intraoperatively yielded eleven nodes, one of which exhibited lymph node metastasis and extranodal extension.

**Figure 2:**
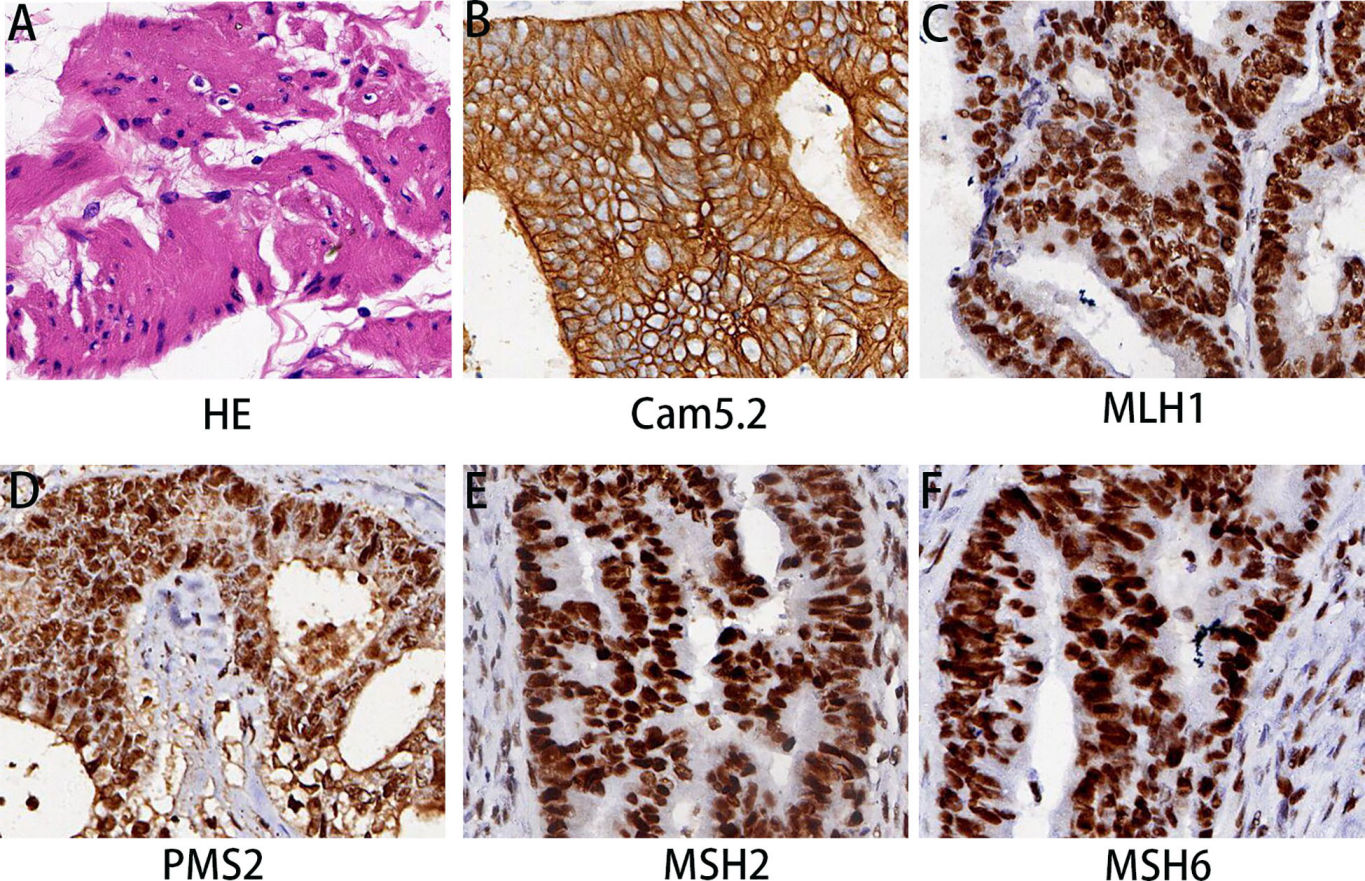
Histopathological findings of rectal mass. **(A)** HE staining revealed differentiated adenocarcinoma in the rectum. **(B–F)** The immunohistochemical examination indicated malignant cells immunoreactive for Cam5.2 **(B)**; MLH1 **(C)**; PMS2 **(D)**; MSH2 **(E)**; and MSH6 **(F)**. Magnification 200×.

**Figure 3:**
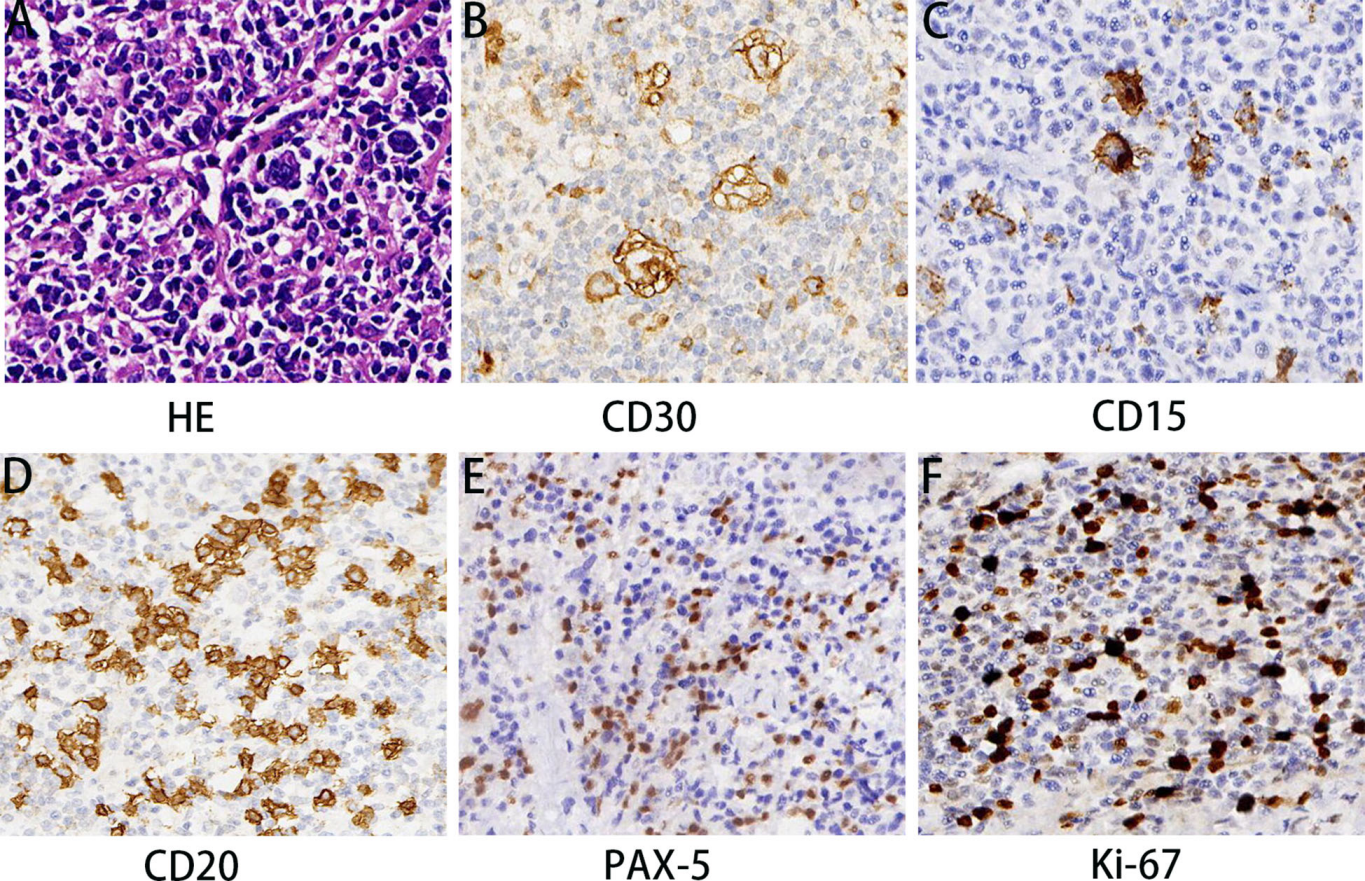
Pathological features of cHL components. HE staining **(A)** demonstrated that cHL component consisted of mixed inflammatory cells and large HRS cells. HRS cells showed positive IHC staining for CD30 **(B)**, CD15 **(C)**, CD20 **(D)**, and PAX-5 **(E)** and the proliferation rate of Ki-67 was approximately 40% **(F)**. Magnification 200×. MRI, Magnetic resonance imaging; MPC, Multiple primary cancers; HE, Hematoxylin and eosin; IHC, Immunohistochemistry; CRC, Colorectal cancer; IBD, Inflammatory bowel disease; cHL, Hodgkin lymphoma; HRS, Hodgkin and Reed-Sternberg; NCCN, National Comprehensive Cancer Network.

Under hematoxylin and eosin (HE) staining, cells were observed to arrange themselves into tubular or nest-like structures, a distinctive characteristic of rectal adenocarcinoma tumor cells (**Figure 2A**). Immunohistochemistry (IHC) results indicated that cancer cells were positive for Cam5.1, CDX-2, and Ki-67 (70%) (**Figure 2B**). Mismatch repair proteins tested included MLH1 (+), PMS2 (+), MSH2 (+), and MSH16 (+), demonstrating no deficiency in mismatch repair proteins (**Figures 2C–F**). In the pelvic lymph nodes, cHL (lymphocyte-rich type) was identified, with no evidence of cancer metastasis. HE staining revealed scattered large mononuclear Hodgkin cells and binuclear or multinuclear Reed-Sternberg cells in the background of mixed inflammatory cells (**Figure 3A**). These large cells exhibited enlarged irregular nuclei and prominent eosinophilic nucleoli. IHC demonstrated positive staining for CD30, CD15, CD20, and PAX5 in the Hodgkin and Reed-Sternberg (HRS) cells (**Figures 3B–E**), as well as decreased expression of Bob-1, and negative expression of CD10 and CyclinD1. The proliferation rate of Ki-67 in the HRS cells was approximately 40% (**Figure 3F**). The final diagnosis of the patient was summarized as follows: 1. Rectal invasive ulcerative moderately differentiated adenocarcinoma, pT3N1M0, stage IIIb; 2. cHL (lymphocyte-rich type) stage IA, classified as low-risk group. Regrettably, the patient did not undergo postoperative adjuvant therapy and was lost to follow-up.

## Discussion

MPC refer to the presence of two or more synchronous or metachronous cancers in the same individual, which was first reported as early as 1921 (8). Three criteria have been proposed by Warren and Gates to characterize MPC as follows: each tumor must exhibit distinct characteristics from the other, displaying well-defined malignancy features. Additionally, the possibility that one is a metastatic lesion originating from the other must be thoroughly ruled out (10). The simultaneous occurrence of rectal adenocarcinoma and lymphoma as MPC is extremely rare. To our knowledge, this case marked the first reported instance of synchronous MPC involving rectal cancer and pelvic Hodgkin lymphoma.

The pathogenesis of MPC has still remained elusive. Several risk factors for this disease have been identified, including genetic factors, immune deficiency and immune escape of cancer cells, accumulation of genetic mutations and abnormal gene expression, as well as the administration of radiotherapy, chemotherapy, and certain drugs (11, 12). Inflammatory bowel disease (IBD) is one of the risk factors for developing CRC (13). Patients with IBD undergoing azathioprine treatment exhibit a substantial four-fold increase in lymphoma risk, suggesting an intriguing association (14). In addition, microsatellite DNA instability, including alterations in chromosome loci, has been identified as a contributing factor to both colon cancer and lymphoma (15). Notably, PD-L1 expression is extremely high in both HL and mismatch repair -deficient colorectal cancer (16, 17).

These are recognized as potential risk factors for the development of MPC involving CRC and lymphoma. However, further research is required to fully understand the exact etiology.

Due to the subtle initial symptoms of CRC, several patients have already been diagnosed with regional lymph node metastasis or even distant organ metastasis at the time of diagnosis (18). Complete radical resection is a potential therapeutic strategy for resectable CRC patients. In our case, extensive imaging investigations revealed multiple enlarged lymph nodes in the left iliac vessel region without any evident abnormalities or contraindications for surgery. Consequently, the patient underwent laparoscopy-assisted abdominoperineal resection combined with mesenteric lymph node dissection, left lateral lymph node dissection, colostomy creation, and abdominal drainage. Postoperative histopathological examination indicated an unexpected finding. The primary lesion was confirmed as rectal adenocarcinoma, while the dissected pelvic lymph nodes showed cHL, indicating a case of atypical MPC. This result further highlights the complexity and challenges in diagnosing MPC.

Distinguishing between metastatic lesions and synchronous tumors represents a crucial diagnostic conundrum. Lymph node metastasis is a prevalent phenomenon in rectal cancer, with a noticeable proportion of patients (approximately 30%) exhibiting locoregional lymph node involvement upon initial diagnosis. Therefore, when imaging reveals multiple enlarged lymph nodes in the left iliac vessel region, the primary consideration typically leans toward the possibility of rectal cancer metastasis, without involvement of concomitant lymphoma. Furthermore, the absence of symptoms associated with cHL, such as fever and night sweats, further diverted our attention from considering the coexistence of lymphoma. This case serves as a reminder that complex and interconnected relationships can exist among diseases, and studying these cases can help to challenge our traditional thinking and broaden our understanding.

Another noteworthy aspect of concern in this case is the challenge of determining whether rectal cancer or Hodgkin lymphoma preceded the other in development or their simultaneous occurrence. In relation to colorectal tumors and lymphoma, Barron and Localio demonstrated that patients with lymphoma have a higher incidence of concurrent colorectal carcinoma (19, 20). The increased risk is mainly attributed to the potential damage inflicted on normal tissues by lymphoma treatment, particularly radiotherapy, elevating the likelihood of developing secondary tumors (21). Secondary malignancies that may emerge following treatment for Hodgkin lymphoma include non-Hodgkin lymphoma, lung cancer, bladder cancer, breast cancer, etc. (22, 23). To date, no study has concentrated on secondary rectal cancer developing from lymphoma. Therefore, early cancer screening is crucial to eliminate this issue.

The management of MPC necessitates collaborative efforts among multidisciplinary medical specialists, encompassing surgical, oncological, radiological, and pathological disciplines (24). Effective cancer treatment mandates careful consideration of key elements, including tumor staging, anatomical location, and the patient’s performance status. Surgery is the cornerstone treatment for patients with operable rectal cancer without surgical contraindications. In regard to elderly patients, age is not a limiting factor for surgery and does not have an impact on survival outcomes (25). In our case, curative surgical intervention was carried out, resulting in postoperative staging of pT3N1M0, indicative of stage IIIb rectal cancer. Unexpectedly, the patient was diagnosed with Hodgkin lymphoma during the pelvic lymph node dissection. According to the National Comprehensive Cancer Network (NCCN) guidelines, adjuvant chemotherapy and radiotherapy are recommended for both stage III rectal cancer and stage I Hodgkin lymphoma. Radiation therapy targeting the pelvic lymph node drainage area is necessitated for both tumors. Additionally, there are similarities in the selection of chemotherapy regimens, and oxaliplatin-based chemotherapy may be considered as an appropriate option. Regrettably, in the present study, the effectiveness of the aforementioned treatments was not assessed due to the patient’s decision to abandon the treatment.

Previous studies demonstrated that patients with MPC tend to have poorer prognoses compared with those with a single primary malignancy (26). The prognosis is influenced by various factors, including tumor stage and biological characteristics of each tumor, treatment response, and patients’ performance status. Due to the limited availability of data and the rarity of concurrent rectal cancer and Hodgkin lymphoma, assessing the prognosis in such cases remains challenging. One limitation of this study is that it is a case report. Hence, it is imperative to gather additional similar cases to establish standardized treatment protocols and assess prognosis more comprehensively.

In conclusion, a rare and intriguing case of MPC was presented involving rectal cancer and Hodgkin lymphoma, providing new clinical data and references to the existing literature on MPC. The underlying mechanisms of the concurrent occurrence of rectal cancer and Hodgkin lymphoma remain elusive, and further exploration is required to elucidate the pathogenesis and develop the effective treatment strategies.

## Data availability statement

The original contributions presented in the study are included in the article/supplementary material. Further inquiries can be directed to the corresponding authors.

## Ethics statement

The studies involving humans were approved by the Ethics and Scientific Committee of Hubei University of Medicine with approval number 2022PR-H002. The studies were conducted in accordance with the local legislation and institutional requirements. The participants provided their written informed consent to participate in this study. The animal studies were approved by the Ethics and Scientific Committee of Hubei University of Medicine with approval number 2022PR-H002. The studies were conducted in accordance with the local legislation and institutional requirements. Written informed consent was obtained from the owners for the participation of their animals in this study. Written informed consent was obtained from the individual(s) for the publication of any potentially identifiable images or data included in this article.

## Author contributions

SL: Funding acquisition, Writing – review & editing, Writing – original draft. HL: Writing – original draft. YD: Conceptualization, Funding acquisition, Writing – review & editing. DZ: Conceptualization, Funding acquisition, Writing – review & editing.
